# The Effects of Cardiac Rehabilitation including Nordic Walking in Patients with Chronic Coronary Syndromes after Percutaneous Coronary Interventions in Elective Mode

**DOI:** 10.3390/medicina59071355

**Published:** 2023-07-24

**Authors:** Rafał Januszek, Bożena Kocik, Wojciech Siłka, Iwona Gregorczyk-Maga, Piotr Mika

**Affiliations:** 1 Department of Cardiology and Cardiovascular Interventions, University Hospital, 30-688 Krakow, Poland; 2 Institute of Clinical Rehabilitation, University of Physical Education in Krakow, 31-571 Krakow, Poland; boz_ka@wp.pl (B.K.); piotr.mika@awf.krakow.pl (P.M.); 3 Jagiellonian University Medical College, 31-008 Krakow, Poland; wojciech.silka@student.uj.edu.pl; 4 Faculty of Medicine, Institute of Dentistry, Jagiellonian University Medical College, 31-155 Krakow, Poland; iwona.g.maga@gmail.com

**Keywords:** coronary artery disease, cardiac rehabilitation, Nordic Walking, percutaneous coronary intervention, physical activity

## Abstract

*Background*: Percutaneous coronary intervention (PCI) in patients with chronic coronary syndrome (CCS) is a worldwide method of coronary revascularisation. The aim of this study was to assess the immediate and long-term effects of Nordic Walking (NW) training added to a standard cardiac rehabilitation programme on physical activity (PA) and capacity and life quality, as well as selected proatherogenic risk factors. *Methods*: The studied group comprised 50 patients (considering exclusion criteria, 40 patients), aged 56–70, with CCS after elective PCI qualified them for a 6-weeks-long cardiac rehabilitation. The follow-up period lasted 4 months, and control visits occurred at 2 and 4 months. The studied patients were randomly divided into two groups: control group—standard cardiac rehabilitation programme and experimental group—standard cardiac rehabilitation programme additionally combined with NW training. *Results*: The cardiac rehabilitation programme in the experimental, compared to the control group, increased intense PA (from 731.43 ± 909.9 to 2740 ± 2875.96 vs. from 211.43 ± 259.43 to 582.86 ± 1289.74 MET min/week) and aerobic efficiency—VO2peak (from 8.67 ± 0.88 to 9.96 ± 1.35 vs. from 7.39 ± 2 to 7.41 ± 2.46 METs), as well as quality of life according to the WHOQOL-BREF questionnaire (from 3.57 ± 0.51 to 4.14 ± 0.36 vs. from 3.29 ± 0.47 to 3.57 ± 0.51 points). The walking distance assessed with the 6-min walk test did not differ between the groups before the beginning of the rehabilitation programme. Both at the I follow-up and II follow-up time points, a significant increase in the walking distance was noted in the control and experimental groups compared to baseline, and the difference between both groups was significant at the end of follow-up (378.57 ± 71.35 vs. 469.29 ± 58.07, *p* = 0.003). Moreover, NW had a positive effect on the modulation within selected biochemical risk factors of atherosclerosis, as well as subjective quality of life and well-being. *Conclusions*: Introducing NW training into the cardiac rehabilitation process proved to be a more effective form of therapy in patients with CCS treated via PCI, as compared to the standard cardiac rehabilitation programme alone.

## 1. Introduction

The beneficial effect of cardiac rehabilitation in patients after acute coronary syndromes (ACSs), impaired left ventricular ejection fraction (LVEF) and other clinical presentations of coronary atherosclerosis (CAD) has been previously demonstrated [1,2,3,4,5,6]. Endothelial dysfunction is one of the many key preclinical manifestations of atherosclerosis [4]. Endothelial cells regulate, among others, vascular tone, and are also involved in the process of haemostasis between pro- and anticoagulant factors [5]. Endothelia produce coagulation inhibitors, including antithrombin III (AT-III). An increased plasma C-reactive protein (CRP) level is an indicator of premature atherosclerosis [6]. The vascular endothelial growth factor (VEGF) contributes to increased vascular permeability and dilation [7]. A significant role here is played by angiopoietin-2 (Ang-2), which, in the absence of VEGF, induces apoptosis of endothelial cells and, thus, causes vascular regression [8]. Of key importance is the fact that pro-inflammatory cytokines affecting endothelial function are among the factors modifiable by physical exercise [9]. Activities in the field of secondary prevention and cardiac rehabilitation are recommended in the treatment of patients with CAD (the highest level of scientific evidence A—recommendation class I) by the European Society of Cardiology (ESC) and the following American societies: American Heart Association, American College of Cardiology and American College of Sports Medicine [10,11]. Significantly popular as a supplement to the second stage of cardiac rehabilitation is the activity of Nordic Walking (NW), i.e., walking in the field with poles [12]. Enrichment of the basic walking technique associated with the use of specially designed poles increases the effectiveness of training by activating the upper body [12]. The European (ESC) and Polish Cardiac Society (PTK), among the recommended activities in cardiac rehabilitation programmes, most often recommend PA, such as walking [13,14]. The ESC recommends undertaking PA with a frequency of 3–5 sessions a week, while emphasising the beneficial effect of daily implementation [13,14]. It is recommended to use moderate-intensity PA of at least 150 min per week, vigorous activity of 75 min per week, or a combination of both in sessions of ≥10 min [14]. The World Health Organization, in order to maintain good physical conditions, also presents its recommendations, which is a minimum of 10,000 steps a day. The most appropriate and most widely promoted questionnaire is the International Physical Activity Questionnaire—IPAQ [15]. An example of a questionnaire used to analyse the area of life in the psychological, physical, social and environmental spheres is the World Health Organization Quality of Life—Bref (WHOQOL-BREF) [16].

The aim of this study is to assess the direct and long-term (first at two months and second at four months) effects of cardiac rehabilitation combined with NW training, conducted among a group of patients with CCS after PCI, in terms of improving PA and fitness, patients’ quality of life and risk factors for the development of atherosclerosis in the coronary arteries. Additionally, the influence was assessed of physical capacity, vascular endothelial function and selected pro-atherosclerotic biochemical indicators, as well as sinus rhythm variability on spiroergometric indicators.

## 2. Materials and Methods

### 2.1. Experimental Group Characteristics

The study included a group of 50 patients (25 men), aged 56–70 years, with CCS and after PCI performed in elective mode. The diagnostic and therapeutic process of CAD was fulfilled as part of the routine diagnostics of the Outpatient Cardiology Clinics and the Department of Cardiology and Cardiovascular Interventions of the University Hospital in Kraków. PCI was performed in accordance with the ESC guidelines for myocardial revascularisation [17,18]. At inclusion to the research project, the pharmacological treatment of patients was stable. Patients from the experimental group were qualified for the programme on the basis of inclusion and exclusion criteria, after signing informed consent. The protocol complied with the 1964 Declaration of Helsinki. The study was performed according to the protocol approved by the Bioethics Committee for Scientific Research at the District Medical Chamber in Kraków (consent No. 90/KBL/OIL). The project received funding from the grant with regard to tasks for the development of young scientists No. 91/MN/KRK/2018 and the funds of the Ministry of Science and Higher Education under the Regional Initiative of Excellence No. 1/BP/RID/2019.

### 2.2. Inclusion Criteria

The inclusion criteria covered the following: stable angina pectoris, history of elective PCI (<3 months), complete coronary revascularisation, LVEF >45% (assessed after complete coronary revascularisation before discharge from hospital or later, before beginning of the rehabilitation programme), consent to be included in the rehabilitation programme, the patient’s condition allowing the treadmill rehabilitation programme to be conducted according to a specific protocol, exercise load on treadmill test before rehabilitation programme ≥5 metabolic equivalents (METs).

### 2.3. Exclusion Criteria

The exclusion criteria covered the following: the presence of contraindications to rehabilitation training; other disease states making it impossible to conduct the rehabilitation programme; life-threatening cardiac arrhythmias; lack of complete revascularization of coronary arteries; advanced atrioventricular block; newly diagnosed atrial fibrillation or flutter; pericarditis or myocarditis; severe symptomatic valvular heart disease, including symptomatic aortic stenosis; severe hypertrophic cardiomyopathy; heart failure according to the New York Heart Association class III and IV (or LVEF < 45%); uncontrolled arterial hypertension; uncontrolled diabetes mellitus; pulmonary hypertension class II, III, IV in accordance with the WHO functional classification; atherosclerosis obliterans of the extremities stage II, III, IV according to the Fontaine classification; acute venous thromboembolism or history within the previous year; history of revascularisation of the coronary and peripheral arteries (PCI, percutaneous transluminal angioplasty [PTA], coronary artery bypass grafting [CABG]) or MI in the last year; respiratory failure; advanced chronic obstructive pulmonary disease or bronchial asthma; acute systemic diseases; active neoplastic disease; the presence of complications at puncture site enabling rehabilitation/walking.

### 2.4. Research Plan

**Figure 1:**
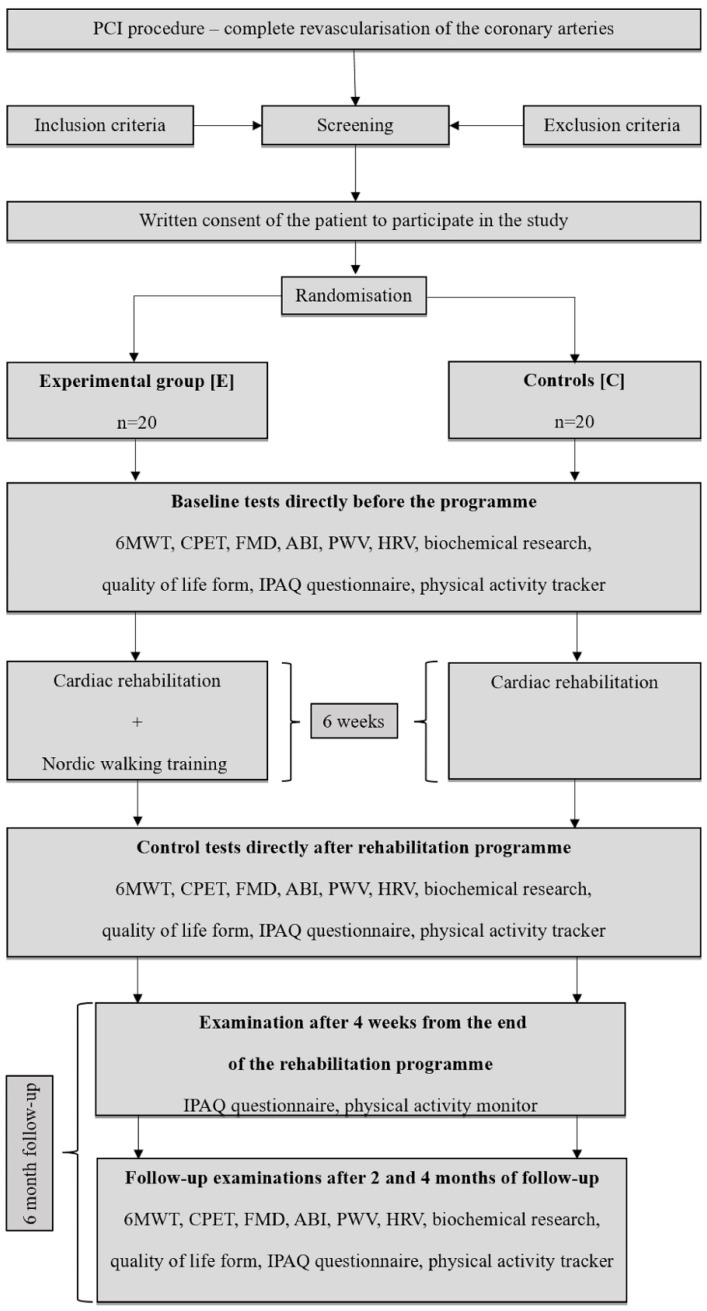
Patient flow chart—6MWT: 6-Minute Walk Test; ABI: ankle-brachial index; CPET: cardiopulmonary exercise testing; FMD: flow mediated dilatation; HRV: heart rate variability; IPAQ: international physical activity questionnaire; PCI: percutaneous coronary intervention; PWV: pulse-waive velocity.

The conducted trial was a randomised experimental clinical study. Taking the established criteria into account, 10 patients were excluded from the study, while the remaining patients were randomised into 2 groups: the experimental group (E) consisting of 20 patients and the control group (C) comprising 20 patients. Patients were recruited between January 2019 and October 2020. The unexpected and difficult time of the COVID-19 pandemic caused difficulties in conducting research among patients from the high-risk group, who, fearing for their health, decided to abandon the project and leave the city for the countryside. In addition, during the study period, some patients were diagnosed with other disease entities, which constituted the criterion for exclusion from the programme. Finally, 13 patients remained in the experimental group and 15 patients in the control (Figure 1).

The time of observation covered a period of 6 months. The programme of cardiac rehabilitation and NW training lasted 6 weeks. Two follow-up periods were specified, respectively after 2 (I F-U) and 4 (II F-U) months from completion of the programme. In addition, a period of 4 weeks from the end of the programme was selected for the assessment.

### 2.5. Study Endpoints

The primary study endpoints included the effect of additional Nordic Walking training implemented as part of a standard cardiac rehabilitation programme in patients treated with PCI in elective mode on improving PA and general fitness. Secondary endpoints of the study included the effect of implementing additional Nordic Walking training on other selected factors, i.e., blood biochemical indices, heart rate variability, cardiopulmonary exercise testing or endothelial function.

### 2.6. Rehabilitation Programme

Based on the result of the exercise test performed on a mechanical treadmill according to the Bruce protocol, patients were qualified to a specific model (A,B) of the second stage of cardiac rehabilitation, considering the model of stratification of cardiac events in accordance with the standards of cardiac rehabilitation [11,12,13,14,17,18]. Patients from the experimental group were included in a standard cardiac rehabilitation programme combined with NW training. In the control group of patients, only a standard cardiac rehabilitation programme was conducted. The same medical recommendations were implemented for both groups of patients in an outpatient setting. Qualification to the experimental and control groups was random [13,14,19,20].

During the cardiac rehabilitation programme, patients performed 5 interval training sessions on different days each week. In addition, patients from the experimental group performed 2 training sessions in the form of NW on the remaining days of the week. 

### 2.7. Cardiac Rehabilitation

Cardiac rehabilitation was fulfilled in the form of interval exercise on a cycle ergometer with the load individually selected according to the heart rate reserve level and the result of the stress test, according to the ESC and PTK guidelines [13,14,17,19]. Interval exercise was performed as follows: five 4 min weighted efforts separated by 1 min periods of no-load riding. The pedalling rhythm was 60 revolutions per minute. The load from baseline during the first interval increased by 5 watts in the second and third intervals, and then decreased similarly by 5 watts in the fourth and fifth ones. In addition, considering individual HRR values, training heart rate ranges were determined for patients in individual rehabilitation schemes. For this purpose, the following principle was used: scheme A—resting heart rate plus 60–80% HRR, scheme B—resting heart rate plus 50–60% HRR. The session of cardiac rehabilitation was preceded by a warm-up and ended with a cool-down. The main part was about 30 min, while the total session duration was around 60 min. The cool-down included a gradual reduction of the load in the final phase of exercise and stretching, breathing and relaxation exercises [17,18].

### 2.8. Nordic Walking Training

The NW training programme lasted for 6 weeks parallel to standard rehabilitation. The patients performed 2 sessions a week, which did not coincide with the sessions of cardiac rehabilitation on a cycle ergometer. The activity was undertaken individually by patients after prior instruction on the NW technique. Patients were provided with the Polar M200 wrist heart rate monitor. The intensity of the training was determined by the heart rate level based on the calculation of the training heart rate in the interval exercise. The patients’ task was to perform a 30 min NW march, causing the heart rate to increase to the designated level. The components of the training session were a warm-up, the main part—walking, and a cool-down phase. The total exercise duration was approximately 60 min [17,18].

### 2.9. Data Collection and Types of Tests

#### 6MWT 6-Minute Walk Test

The test was performed in accordance with the indications and contraindications, as well as recommended safety requirements and the availability of appropriate equipment [21].

### 2.10. Objective and Subjective Assessment of PA

PA was conducted using a wrist heart rate monitor (Polar M200 GPS Running Watch, Polar Electro Oy, Finland). Summarised data regarding the patients’ PA for the designated time interval included information related to the measured activity time, the number of recorded steps, the distance travelled based on the number of steps, and the number of calories burned. The measurements were compared with the IPAQ questionnaire completed by the patients and covered a period of 7 days [15]. PA was subjectively assessed using the IPAQ—Polish short version, according to the published protocol [22]. 

### 2.11. CPET—Cardiopulmonary Exercise Test

The ECG stress test with direct breathing gas analysis was performed on an ECG stress treadmill (BTL Limited Industries, Boston, MA, USA). The standard Bruce protocol [18] with escalating and computer-controlled loading was used in accordance with current guidelines [13,17,18]. Throughout the observation period, the MetaLyzer 3B-R2 ergospirometer (Cortex, Germany) was used to analyse selected respiratory indices. The following indicators were assessed: VO2 [L/m]—global oxygen consumption; VO2 [mL/min/kg]—relative oxygen consumption; HR [L/min]—heart rate; VO2/HR [mL/beat]—oxygen pulse; VE [L/min]—minute ventilation; VE/VO2 [L/mL]—oxygen ventilation equivalent; MET—metabolic equivalent.

### 2.12. FMD, ABI and PWV Assessment

FMD assessment was performed according to the previously published protocol [4,9].

Other measurements (ABI, PWV) were performed in an adapted room with standardised rules and in the supine position after the patients underwent a 10 min adaptation period [23,24]. For this purpose, the Boso apparatus (ABI System 100 + PWV, BOSCH + SOHN, Gerlingen, Germany) was used. 

### 2.13. Biochemical Analyses

The following biochemical tests were performed: lipidogram (TC [total cholesterol], LDL-C [low-density lipoprotein cholesterol], HDL-C [high-density lipoprotein cholesterol], TGL [triglycerides]), pro-inflammatory marker (CRP), markers of thrombosis (Fibrinogen, Antithrombin III), markers of angiogenesis (VEGF, Ang-2), a marker of heart failure (NT-proBNP [N-terminal natriuretic propeptide type B]). The material for the study was collected in the morning, with a 14 h break from the last meal. Patients were instructed to limit their consumption of high-fat meals and alcohol, and to refrain from smoking and undertaking increased PA in the 2–3 days preceding the study. In order to determine the biochemical parameters, the patient’s blood was collected from the vein of the forearm after a 15 min rest. Particular methods of biochemical determinations included TC, LDL-C, HDL-C, TGL—enzymatic method, colourimetric method, determinations made on the Cobas Pro, C-503 analyser (Roche Diagnostics, Indianapolis, IN, USA); CRP—immunoturbidimetric method, determinations made on the Cobas Pro, C-503 analyser (Roche Diagnostics, Indianapolis, IN, USA); Fibrinogen—Clauss method, using the Multifibren reagent (Siemens Healthcare Diagnostic, Erlangen, Germany), determinations made on the AtellicaCoag 360 coagulation analyser (Siemens Healthcare Diagnostic, Erlangen, Germany); Antithrombin III—chromogenic method, using the INNOVANCE Antitrombin test (Simems Healthcare Diagnostic, Erlangen, Germany), determinations made on the AtellicaCoag 360 coagulation analyser (Siemens Healthcare Diagnostic, Erlangen, Germany); VEGF—xMAP Luminex method using the phenomenon of fluorescence (Luminex, Austin, TX, USA); Angiopoietin-2—Luminex xMAP fluorescence method (Luminex, Austin, TX, USA); and NT-proBNP—immunological method based on electrochemiluminescence (ECLIA), determinations carried out on the Cobas Pro, E-801 analyser (Roche Diagnostics, Indianapolis, IN, USA).

### 2.14. HRV—Heart Rate Variability Assessment

Heart rate variability analysis was performed using the Biopac Systems device (Inc. MP 150, Santa Barbara, CA, USA), using an ECG100C electrocardiogram amplifier. 

### 2.15. Assessment of Patients’ Quality of Life

The assessment of the quality of life was carried out using the World Health Organisation Quality of Life—Bref (WHOQOL-BREF) questionnaire [25].

### 2.16. Statistical Analysis

The normality of distribution of variables in groups was checked using the Shapiro–Wilk test. Non-parametric tests were used to analyse differences between the examined variables. The Kruskal–Wallis rank ANOVA test was used to evaluate the significance of differences in the dependent variables. Dunn’s test was used for post hoc evaluation. The Mann–Whitney U test was applied for inter-group comparisons. Differences were considered statistically significant if the test probability was lower than the assumed level of significance (*p* < 0.05). Data are presented as means (x) and standard deviations (SDs). STATISTICA12.0 PL was used for statistical calculations.

## 3. Results

The research programme was completed by 28 people, including 6 women and 22 men. There were 13 patients in the experimental group (2 women and 11 men) and 15 controls (4 women and 11 men). Before being included in the rehabilitation programme, all patients underwent full revascularization of the coronary arteries in the case of hemodynamically significant stenoses, which, in case of doubts based on angiography, were verified invasively by measuring the partial coronary flow reserve. Data on the arteries that were revascularized are presented in Table 1. The clinical characteristics and pharmacotherapy before entering the study are presented in Table 1.

### 3.1. Assessment of Exercise Tolerance

#### 3.1.1. Six-Minute Walk Test

The walking distance assessed with the 6MWT test did not differ between the groups before the beginning of rehabilitation. Both in the IF-U and II F-U periods, an increase in the walking distance was noted in both groups compared to baseline (Table 1).

#### 3.1.2. Objective and Subjective Assessment of PA

The PA of the patients expressed in the number of hours and covering the period of 7 days did not change significantly in any of the groups. There were also no significant differences between the study groups. The PA of the patients expressed in the number of steps, covering the period of 7 days, did not differ between the groups before beginning rehabilitation. After rehabilitation and after 4 weeks from its end, significantly higher PA levels were found among experimental group patients compared to controls (Table 2).

Vigorous PA of patients assessed with the IPAQ questionnaire, covering a period of 7 days, did not differ between the groups at baseline. After rehabilitation and after 4 weeks following the completion of rehabilitation, only patients from the experimental group presented significantly higher vigorous PA levels in comparison to baseline values. The observed change was greater among the experimental group compared to the controls (Table 2). Those and other indices measuring PA are presented in Table 2.

### 3.2. Cardiopulmonary Exercise Testing

Before the rehabilitation, the peak oxygen uptake among the experimental group was, on average, 2.03 [L/min], while in the control group, it was slightly lower and amounted to 1.74 [L/min]. In the I and II F-U period, the VO2peak level in the experimental group increased significantly to 2.28 and 2.24 L/min, respectively, while in the control group, it did not change (Table 3).

### 3.3. FMD, ABI and PWV

The FMD value did not differ between the groups at baseline and did not change significantly after rehabilitation. However, the groups differed significantly after the end of rehabilitation and after the I F-U period (Table 4).

The value of the ABI for the right and left lower limbs did not differ between the groups at baseline and did not change throughout the programme. The cervical–femoral pulse wave velocity index (cfPWV_calc), calculated from baPWV, did not differ between the groups before the start of rehabilitation. After rehabilitation, in the experimental group, the value of the cervical–femoral pulse wave velocity index was significantly lower than the level before rehabilitation. During this period, a significant difference between the groups was also observed (Table 4).

### 3.4. Selected Biochemical Indices

#### 3.4.1. Lipid Profile

The TC serum concentration did not differ between the groups at baseline. After rehabilitation, a statistically significant difference in the increase of TC was observed in the control group. Also, a significant difference between the groups was visible after the rehabilitation period. These results and the impact of physical exercise on other lipid profile fractions are presented in Table 4.

#### 3.4.2. Pro-Inflammatory Markers

The CRP concentration did not differ between the groups at baseline. In the experimental group, post rehabilitation, a significant decrease in the concentration of this parameter was observed. Also, a significant intergroup difference, resulting from a lower CRP concentration in the experimental group, was visible after the rehabilitation period (Table 4).

#### 3.4.3. Fibrinogen and Antithrombin III

The fibrinogen concentration did not differ between the groups at baseline. After rehabilitation in the experimental group, the concentration of fibrinogen decreased significantly. A significant inter-group difference was also observed during this period (Table 4). Antithrombin III (AT III) activity did not differ between the groups at baseline. After rehabilitation, in both groups, no significant changes were found at subsequent time points within the observed groups and between them (Table 4).

#### 3.4.4. Markers of Angiogenesis

The VEGF concentration did not differ between the groups at baseline. In both groups, decreasing VEGF concentrations were after the I F-U period in the control group and after the II F-U period in the experimental group (Table 4). The concentration of Ang-2 did not differ between the groups at baseline. After rehabilitation in the experimental group, the concentration of Ang-2 was significantly greater in that group than in the control (Table 4).

#### 3.4.5. B-Type Natriuretic Peptide

The NT-proBNP concentration did not differ between the groups at baseline. After rehabilitation in the experimental group, a significant decrease in the concentration of NT-pro BNP was observed (Table 4).

### 3.5. Assessment of Heart Rate Variability

The activity of the sympathetic and parasympathetic nervous systems, as well as the sympathetic–parasympathetic balance index, did not differ between the groups before beginning rehabilitation and did not change significantly in either group at subsequent time points (Table 3).

### 3.6. Assessment of Patients’ Quality of Life

The patients’ quality of life, assessed with the WHOQOL-BREF questionnaire, did not differ between the groups at baseline. After the rehabilitation period, improvement in the patients’ quality of life was observed in the experimental group (Table 5).

## 4. Discussion

Considering the results of the current study, the proposed cardiac rehabilitation programme with the additionally implemented NW training and regular rehabilitation intervention, not including this form of training, led to an increase of intense PA, aerobic efficiency and quality of life. It also had a positive effect on the modification of selected biochemical pro-atherosclerotic blood markers. The observed beneficial changes in the group of patients with CCS after PCI were noted both immediately after rehabilitation and in the follow-up period. The effects of the discussed changes were more visible in the cardiac rehabilitation programme with the additionally implemented NW training.

Regular PA is prophylactic in cardiovascular diseases and, independently of other factors, reduces the likelihood of CAD. The beneficial effect of PA is directly reflected in the improvement of vascular endothelial function, as well as the prothrombotic state, lipid profile, proinflammatory state and arterial pressure [26]. The advantageous influence of regular PA associated with haemodynamic changes in the body primarily results in increasing the physical capacity of patients [27]. Systematic physical exercise not only leads to a reduction in pro-atherogenic markers, but also contributes to the improvement of myocardial perfusion. The positive effects of PA translate into general health and the degree of prophylaxis observed at a later stage, in the prevention of cardiovascular diseases [14,28,29].

The recently published trial ISCHEMIA, which analysed the impact of revascularization in patients with chronic coronary syndromes, demonstrated that invasive management did not lower all-cause mortality at 4 years in any ischemia or CAD subgroup [30,31]. Therefore, it is important to evaluate other methods of improving the functioning of patients with CAD undergoing elective percutaneous revascularization procedures. One of them is the aforementioned physical rehabilitation. However, standard cardiological rehabilitation programmes can be enriched with other motor activities, such as NW. The results of the study by Girold et al. [12] on patients with cardiovascular diseases, including ACSs, allowed confirming that patients participating in a 4-week training programme, enriched with NW activity, achieved significant improvement in walking distance, assessed using the 6MWT, when compared to patients walking without poles. In the current study, we noted that among patients from the experimental group, the PA of patients, defined by the number of registered steps and the distance travelled, was significantly greater immediately after the rehabilitation period and 4 weeks after its completion, compared to the control group. In light of our own research, analysing the periods selected above, a significant increase in the number of registered steps was observed in the experimental group of patients, by approximately 134% and 114%, respectively, in relation to the 70,000 steps per week recommended by WHO. Also, meta-analysis of controlled and randomised clinical trials on the usability of NW in rehabilitation of patients with cardiovascular diseases performed by Cugusi et al. determined that, in CAD, significant differences between NW plus conventional cardiac rehabilitation and conventional cardiac rehabilitation alone were found in exercise capacity and dynamic balance favouring NW plus conventional cardiac rehabilitation; in peripheral artery disease, larger changes in exercise duration and oxygen uptake were observed following NW compared with controls; in heart failure, no significant differences were found between NW and conventional cardiovascular rehabilitation or usual care for peak VO2 and functional mobility, while in post-stroke survivors, functional mobility was significantly higher following treadmill programmes with poles rather than without [32]. The use of NW in patients after complete revascularization of the coronary arteries is described in the literature, but studies with the use of NW in patients with incomplete or no coronary artery revascularization have not been published to our knowledge. Analysing the results of our study, the improvement of motor skills was present only after a longer follow-up period in the experimental group; it may be related to the influence of developing the habit of additional physical activities, such as NW, in the later period and a delayed response to them. On the other hand, the fact that the main improvement was physical activity expressed in the 6-min walk test, and not the overall weekly physical activity, may be explained by the fact that the activity itself was already at a high level for patients from this group of patients, and only its intensity changed.

The research method used in our project, combining the assessment of PA based on the above-mentioned parameters, using a Polar M200 wrist heart rate monitor in patients after PCI, seems to be an innovative approach, as there is no similar information in the analysed literature. Another important issue presented in the study by Peterson et al. [33] is the effectiveness of promoting and monitoring PA in patients following PCI. With regard to the considerations above, it is crucial to encourage patients to monitor and achieve their daily PA goals. Similar results evaluating PA using the IPAQ questionnaire and covering a group of cardiac patients, including those after PCI, were presented in the study by Soares et al. [34]. The use of self-monitoring systems, in the form of wrist-based heart rate monitors, activated and set the patients from the experimental group a new, realistic objective every day.

From the current analysis, it was found that in the experimental group of patients, the VO2peak value [L/min], in global terms, was significantly higher immediately after rehabilitation and 4 months following its completion, compared to the baseline value. Even a small increase in VO2peak after rehabilitation increases the exercise capacity of the patients form the experimental group and improves the risk profile of CAD [35,36]. A similar relationship was observed in the study by Belardinelli et al. [37]. A slight improvement in exercise tolerance after a 6-week cardiac rehabilitation programme in patients after PCI and CABG was also observed in the study by Jelinek et al. [38]. The effect of a 3-month training programme on exercise tolerance in a group of patients following PCI and CABG was also described in the study by Lan et al. [39]. It was noted that in both groups of patients (PCI/CABG), there was an increase in the post-training value of the VO2peak parameter [mL/min/kg] by 14.6% and 32.8%, respectively. In the comparison of brisk walking without the use of poles and NW, it is estimated that the increase in the energy expenditure parameter, expressed in VO2, ranges from 11 to 23% [40]. The value of the maximum minute oxygen uptake in the group of healthy people expressed in metabolic equivalent units is around 13 MET, while in the group of people with CAD, the value of VO2max is much lower and even reduced to 4 MET (14 mL/kg/min). Comparing the effects of NW activity with those of training in the form of ordinary walking in the study by Church et al. [41], a significant increase in the peak heart rate was noted in the study groups of men and women. The authors suggest that practicing NW activity contributes to an increase in VO2peak, energy expenditure and heart rate, without significantly increasing the subjective feeling of physical exertion. The results of our analysis are consistent with those obtained in the study by Lan et al. [39], who evaluated cardiorespiratory parameters, including oxygen pulse, in patients undergoing PCI and CABG. In the research conducted by Baek et al. [42], the effects of NW and walking without the use of poles in a group of healthy men were compared, with the assessment of respiratory indices taking into account, among others, the minute ventilation rate of the lungs. The evaluation of this indicator showed its increase (+17.0%) in men subjected to NW activity. The suggested basis of these changes probably resulted from the additionally implemented NW activity [42].

In light of our research, only among patients from the experimental group, after the period of rehabilitation and after 2 months from its completion, improvement was noted in FMD, compared to the control group. In our opinion, the main reason for the improvement of endothelial function, estimated as the dilation of the brachial artery in this group of patients, was the activation of larger muscle groups, which could be directly related.

Among the important indicators of frequency analysis, the power component of the low- (LF) and high-frequency spectrum (HF) deserve attention. Sympathetic activation associated with an increase in the LF amplitude is observed in stressful situations or recent MI, in contrast to the increase of the HF component occurring in the case of increased vagal tone [43,44]. Although our study did not show any relationship between rehabilitation and NW with the activity of the sympathetic and parasympathetic systems, which may be explained by the short duration of the study or the small group of patients, a number of studies have shown such a relationship [45]. Increased activity of the sympathetic nervous system, coexisting with decreased activity of the vagus nerve, predisposes one to the occurrence of malignant arrhythmias, sudden cardiac death, myocardial hypertrophy or the aforementioned arterial hypertension and progression of atherosclerotic lesions [46].

General questionnaires most fully define the concept of HRQOL by measuring the quality of life in a comprehensive manner and in a wide range [47]. The lack of an appropriate rehabilitation model and preventive procedures leads to disability and social and family isolation. The result of neglect is insufficient control of CAD risk factors, a significant number of deaths, MIs and other serious cardiovascular complications [29]. Significantly better outcomes resulting from the improvement of quality of life related to the health of patients could be seen among people undergoing cardiac rehabilitation. Analysing our research, a significant increase in the value of this parameter was noted in the experimental group after the rehabilitation period. In the control group, these changes were observed only after 4 months following completion of the programme. Such a result could have been influenced by a number of factors, and one of them is certainly NW itself, which is associated with outdoor activity, contact with the environment, nature and activating other muscle groups. All these factors can improve mood, positive perception of society and faster return to life before the procedure. With regard to the cited publications, it also turns out that comorbidities have a significant impact on the assessment of patients’ quality of life [18]. It is worth noting that in CAD, there are often dysfunctions in the cognitive–emotional area, manifested by depression, anxiety and increased stress. It has been observed that depression may contribute to the lack of improvement in the quality of life among patients, even after successful PCI [40].

## 5. Strengths and Limitations of the Study

The main objection to the presented work is the much smaller-than-planned number of patients, which was forced by the period of the COVID-19 pandemic, which began shortly after implementation of the study. The strength of the presented study is a very broad insight into a number of potential mechanisms related to the improvement of exercise tolerance in the group of patients with chronic coronary syndrome undergoing elective percutaneous coronary revascularization procedures as a result of cardiac rehabilitation, depending on the model used and the presence of NW. The relationship between selected methods of rehabilitation and angiogenesis, coagulation, pro-inflammatory markers, endothelial function and vascular stiffness and, at the same time, assessment of mental state and physical fitness indicators, including the circulatory and respiratory systems, were assessed. An additional factor that could have influenced the results of the study is the fact that NV was an additional activity that was undertaken on those days of the week when there was no rehabilitation. It can, therefore, be assumed that any other physical effort conducted with a similar intensity on those days of the week when a standard cardiac rehabilitation programme was not conducted would have a similar effect. However, NW is a specific type of activity that engages larger muscle groups and is relatively simple to perform and does not require special weather, equipment, environment or place of residence.

## 6. Conclusions

In light of our own research, the compared rehabilitation programmes conducted in the group of patients with chronic coronary syndromes after elective PCI significantly contributed to beneficial changes in terms of improving PA and fitness, patients’ quality of life and the profile of selected risk factors in the development of atherosclerosis within the coronary arteries. The introduction of NW training into the cardiac rehabilitation programme, in many aspects, observed both immediately after rehabilitation and in the form of its long-term effects, turned out to be a more effective form of therapy compared to implementing the regular rehabilitation programme alone.

## Figures and Tables

**Table 1:** Baseline clinical characteristics and pharmacotherapy.

Indices	Mean/Number	±SD/(%)	Experimental Group (*n* = 13)		Controls (*n* = 15)		*p*-Value
			Mean/Number	±SD/(%)	Mean/Number	±SD/(%)	
Age, years	63	7.38	60	7.31	64	6.07	0.82
Gender, males	22	(79%)	11	(85%)	11	(73%)	0.79
Height, cm	174	9.33	177	5.92	171	10.75	0.08
Weight, kg	86.5	14.44	86	9.74	87	17.9	0.85
Body mass index [kg/m^2^]	28,6	3.65	27.4	2.44	29.6	4.26	0.11
Smoking	24	86%	12	92%	12	80%	0.69
Active smoking	12	43%	8	62%	4	27%	0.06
Length of smoking, years	24	15.01	26	14.17	23	16.02	0.6
Arterial hypertension	26	93%	11	85%	15	100%	0.11
Hypercholesterolemia	22	79%	9	69%	13	87%	0.26
Prior PCI	12	43%	8	62%	4	27%	0.06
Prior myocardial infarction	10	363%	6	46%	4	27%	0.28
Prior CABG	0	0%	0	0%	0	0%	NA
LVEF, %	57	8.78	59	8.74	56	8.93	0.37
ASA	28	100%	13	100%	15	100%	NA
Clopidogrel	16	57%	5	38%	11	73%	0.06
Ticagrelor	12	43%	8	62%	4	27%	0.06
Beta-blocker	22	79%	9	69%	13	87%	0.26
ACE-I/ARB	28	100%	13	100%	15	100%	NA
Statin	22	79%	9	69%	13	87%	0.26
RCA	10	35.7%	4	30.8%	6	40%	0.91
Cx	5	17.8%	2	15.4%	3	20%	0.75
LAD	10	35.7%	5	38.4%	5	33.3%	0.91
LVB	1	3.6%	1	7.7%	0	0%	0.27
>2 arteries	2	7.2%	1	7.7%	1	6.7%	0.91

ACE-I: angiotensin converting enzyme inhibitor; ASA: acetyl-salicylic acid; ARB: angiotensin receptor blocker; CABG: coronary artery bypass grafting; LVEF: left ventricle ejection fraction; PCI: percutaneous coronary intervention.

**Table 2:** Indices of physical activity.

Time Points	Control	Experimental Group	*p*-Value
6-min walk test [m]			
At baseline	341.57 ± 78.37	388.86 ± 76.67	0.24
After rehabilitation	414.29 ± 48.31 *	447.86 ± 73.69 *	0.32
2-month follow-up	412.86 ± 42.86 **	462.86 ± 52.32 **	0.017
4-month follow-up	378.57 ± 71.35 ***	469.29 ± 58.07 ***	0.003
Physical activity [hours/week]			
At baseline	22.71 ± 19.75	32.14 ± 15.65	0.28
After rehabilitation	35.00 ± 8.86	43.86 ± 15.18	0.87
1-month follow-up	32.14 ± 17.37	40.43 ± 12.61	0.66
2-month follow-up	31.71 ± 16.17	36.57 ± 13.99	0.47
4-month follow-up	27.28 ± 16.33	40.00 ± 17.17	0.32
Physical activity [number of steps/week]			
At baseline	46,884.43 ± 38,024.49	65,372.29 ± 33,542.4	0.013
After rehabilitation	61,897.71 ± 31,223.02	94,179.14 ± 43,860.88	0.036
1-month follow-up	58,796.43 ± 49,346.99	79,628.86 ± 38,109.87	0.022
2-month follow-up	56,406.86 ± 44,082.93	74,910.14 ± 42,655.58	0.12
4-month follow-up	45,202.43 ± 45,777.24	75,430.14 ± 50,784.24	0.12
Physical activity [km/week]			
At baseline	26.96 ± 20.15	39.63 ± 21.64	0.013
After rehabilitation	35.63 ± 22.48	57.19 ± 27.77	0.013
1-month follow-up	33.93 ± 30.48	48.09 ± 24.27	0.022
2-month follow-up	31.38 ± 24.92	46.02 ± 27.58	0.08
4-month follow-up	25.98 ± 26.55	46.15 ± 31.48	0.056
Physical activity [kcal/week]			
At baseline	12,973.29 ± 3457.75	14,186.57 ± 4030.65	0.17
After rehabilitation	15,639.43 ± 3127.21	17,285.86 ± 2461.28	0.12
1-month follow-up	15,007 ± 3555.66	17,023.29 ± 1979.22	0.084
2-month follow-up	14,690.43 ± 3751.89	16,459.71 ± 1966.33	0.24
4-month follow-up	13,487 ± 4824.15	16,428.71 ± 3088.83	0.036
Intense physical activity [MET min/week]			
At baseline	211.43 ± 259.43	731.43 ± 909.9	0.42
After rehabilitation	582.86 ± 1289.74	2740 ± 2875.96 *	0.029
1-month follow-up	765.71 ± 1051.87	2271.43 ± 1678.09 **	0.013
2-month follow-up	560 ± 638.46	1114.29 ± 1570.56	0.66
4-month follow-up	1120 ± 2396.3	1954.29 ± 1837.11 ****	0.32
Moderate physical activity [MET min/week]			
At baseline	1388.57 ± 1823.57	1420 ± 1041.01	0.28
After rehabilitation	2045.71 ± 2582.99	2571.43 ± 1816.4	0.47
1-month follow-up	1494.29 ± 1856.41	2225.71 ± 1530.36	0.24
2-month follow-up	1722.86 ± 1895.89	2474.29 ± 1653.93	0.32
4-month follow-up	742.86 ± 1031.89	2160 ± 1635.9	0.013
Walking [MET min/week]			
At baseline	1295.86 ± 1334.5	2051.71 ± 1501.86	0.017
After rehabilitation	2253.43 ± 1445.84 *	2012.79 ± 1265.1	0.47
1-month follow-up	905.51 ± 841.51	3246.29 ± 2961.51	0.002
2-month follow-up	1145.57 ± 1141.2	3230.29 ± 2524.89 ***	0.006
4-month follow-up	872.57 ± 866.76	2015.36 ± 1162.57	0.005
Sitting [min/weekdays]			
At baseline	1328.57 ± 662.66	935.71 ± 396.83	0.036
After rehabilitation	1200 ± 1012.23	842.86 ± 333.32	0.73
1-month follow-up	878.57 ± 541.97 **	1014.29 ± 743.54	0.47
2-month follow-up	728.57 ± 548.32 ***	875 ± 847.73	0.73
4-month follow-up	1028.57 ± 939.25 ****	1071.43 ± 623.8	0.32

*—when *p* < 0.05 for comparison between two indices: after rehabilitation and at baseline, **—when *p* < 0.05 for comparison between two indices: 1-month follow-up and at baseline. ***—when *p* < 0.05 for comparison between two indices: 2-month follow-up and at baseline. ****—when *p* < 0.05 for comparison between two indices: 4-month follow-up and at baseline.

**Table 3:** Cardiopulmonary exercise testing and heart rate variability assessment.

Time Points	Control	Experimental Group	*p*-Value
Cardiopulmonary exercise testing			
V02peak [L/min]			
At baseline	1.74 ± 0.54	2.03 ± 0.34	0.64
After rehabilitation	1.75 ± 0.63	2.29 ± 0.19 *	0.012
2-month follow-up	1.75 ± 0.52	2.28 ± 0.31	0.023
4-month follow-up	1.64 ± 0.38	2.24 ± 0.288 ***	0.011
VO2peak [mL/min/kg]			
At baseline	23.00 ± 2.00	24.29 ± 2.13	0.078
After rehabilitation	19.86 ± 4.45	27.14 ± 2.91	0.006
2-month follow-up	20.57 ± 3.72	26.86 ± 2.51	0.034
4-month follow-up	21 ± 3.37	26.57 ± 2.77	0.041
VO2peak [s]			
At baseline	340.29 ± 123.3	418.57 ± 38.22	0.33
After rehabilitation	343.43 ± 133.23	514.43 ± 90.41	0.006
2-month follow-up	405.86 ± 100.09 **	515 ± 57.96 **	0.014
4-month follow-up	454 ± 99 ***	526.71 ± 59.2 ***	0.024
Heart rate [1/min]			
At baseline	122.57 ± 9.35	127.43 ± 11.04	0.12
After rehabilitation	127.86 ± 14.03	127.43 ± 7.88	0.67
2-month follow-up	125.71 ± 8.32	129.14 ± 12.61	0.43
4-month follow-up	122.71 ± 8.75	130.86 ± 14.59	0.08
VO2/Heart rate [mL/beat]			
At baseline	14.29 ± 4.32	17.14 ± 2.96	0.34
After rehabilitation	14 ± 6.13	18 ± 1.92	0.031
2-month follow-up	13.86 ± 4.55	17.86 ± 2.25	<0.04
4-month follow-up	14.57 ± 3.41	17.14 ± 2.03	0.71
Minute ventilation (VE) [L/min]			
At baseline	65.27 ± 16.7	73.57 ± 14.93	0.21
After rehabilitation	65.67 ± 20.38	84.06 ± 14.48 *	0.03
2-month follow-up	68.59 ± 20.73	83.89 ± 15.02	0.57
4-month follow-up	69.84 ± 20.89	86.7 ± 10.69 ***	0.01
Minute ventilation (VE)/V02 [L/mL]			
At baseline	34.97 ± 4.7	35.2 ± 5.18	0.98
After rehabilitation	35 ± 4.42	34.43 ± 5.19	0.87
2-month follow-up	36.29 ± 5.42	34.61 ± 5.05	0.65
4-month follow-up	34.8 ± 4.22	36.4 ± 3.9	0.68
VO2peak [MET]			
At baseline	7.39 ± 2	8.67 ± 0.88	0.32
After rehabilitation	7.41 ± 2.46	9.96 ± 1.35 *	0.003
2-month follow-up	8.36 ± 1.7 **	10.5 ± 0.86 **	0.013
4-month follow-up	8.97 ± 1.81 ***	10.11 ± 1.16 ***	0.33
Heart rate variability assessment			
Sympathetic activity of the nervous system			
At baseline, m	0.56 ± 0.2	0.54 ± 0.18	0.32
After rehabilitation, m	0.47 ± 0.18	0.46 ± 0.17	0.94
2-month follow-up, m	0.48 ± 0.13	0.53 ± 0.2	0.80
4-month follow-up, m	0.44 ± 0.12	0.49 ± 0.14	0.32
Parasympathetic activity of the nervous system			
At baseline	0.44 ± 0.2	0.46 ± 0.18	0.32
After rehabilitation	0.53 ± 0.18	0.54 ± 0.17	0.94
2-month follow-up	0.52 ± 0.13	0.47 ± 0.2	0.80
4-month follow-up	0.56 ± 0.12	0.51 ± 0.14	0.32
Sympathetic and parasympathetic activity ratio			
At baseline	1.74 ± 1.12	1.43 ± 0.77	0.32
After rehabilitation	1.34 ± 1.44	1.22 ± 1.26	0.94
2-month follow-up	1.09 ± 0.72	2.1 ± 2.79	0.80
4-month follow-up	0.92 ± 0.69	1.13 ± 0.69	0.32

*—when *p* < 0.05 for comparison between two indices: after rehabilitation and at baseline. **—when *p* < 0.05 for comparison between two indices: 1-month follow-up and at baseline. ***—when *p* < 0.05 for comparison between two indices: 2-month follow-up and at baseline.

**Table 4:** Endothelial function, pulse wave velocity and selected biochemical indices.

Time Points	Control	Experimental Group	*p*-Value
Flow-mediated dilatation [%]			
At baseline	4.98 ± 3.68	6.39 ± 4.67	0.07
After rehabilitation	4.75 ± 2.24	7.06 ± 2.48	0.01
2-month follow-up	3.21 ± 1.71	6.96 ± 2.57	0.008
4-month follow-up	3.7 ± 0.95	5.36 ± 5.01	0.09
baPWV—right side [m/s]			
At baseline	11.94 ± 1.25	11.11 ± 0.74	0.0003
After rehabilitation	12.94 ± 2.46	10.96 ± 1.16	0.01
2-month follow-up	12.34 ± 1.54	11.27 ± 1.82	0.056
4-month follow-up	12.37 ± 2.11	11.54 ± 1.32	0.022
baPWV—left side [m/s]			
At baseline	12.53 ± 1.26	11.59 ± 1.7	0.004
After rehabilitation	13.37 ± 1.02 *	11.17 ± 1.76	0.001
2-month follow-up	13.57 ± 1.64 **	11.79 ± 2.68	0.013
4-month follow-up	13.17 ± 1.39	11.94 ± 2.02	0.013
cfPWV_calc [m/s]			
At baseline	8.26 ± 1.03	7.46 ± 1.32	0.003
After rehabilitation	9.37 ± 1.65	7.16 ± 1.32 *	0.0003
2-month follow-up	9.17 ± 1.11	7.64 ± 2.17	0.008
4-month follow-up	8.81 ± 1.38	7.89 ± 1.93	0.013
Total cholesterol [mmoL/L]			
At baseline	3.46 ± 0.4	3.8 ± 1.01	0.55
After rehabilitation	4.46 ± 0.94 *	3.97 ± 1.96	0.02
2-month follow-up	4.66 ± 1.3 **	4.11 ± 1.79	0.56
4-month follow-up	4.41 ± 0.72 ***	4.11 ± 1.26	0.61
Low-density lipoprotein cholesterol [mmoL/L]			
At baseline	1.64 ± 0.48	1.61 ± 0.89	0.86
After rehabilitation	2.03 ± 0.72	1.94 ± 1.19	0.56
2-month follow-up	2.44 ± 1.25	1.79 ± 0.6	0.09
4-month follow-up	2.37 ± 0.75 ***	2.11 ± 0.86	0.47
High-density lipoprotein cholesterol [mmoL/L]			
At baseline	1.04 ± 0.21	1.12 ± 0.36	0.34
After rehabilitation	1.17 ± 0.37	1.21 ± 0.34	0.65
2-month follow-up	1.24 ± 0.23 **	1.21 ± 0.4 **	0.39
4-month follow-up	1.21 ± 0.3	1.17 ± 0.37	0.58
Triglycerides [mmol/l]			
At baseline	1.94 ± 1.55	2.35 ± 2.83	0.28
After rehabilitation	3.37 ± 4.39 *	1.8 ± 1.93 *	0.006
2-month follow-up	2.19 ± 0.57 **	2.45 ± 3.72	0.014
4-month follow-up	1.82 ± 0.45	1.83 ± 1.92	0.89
C-reactive protein [mg/L]			
At baseline	4.95 ± 2.75	6.14 ± 6.36	0.09
After rehabilitation	3.31 ± 2.67	1.32 ± 1.36 *	0.007
2-month follow-up	1.92 ± 0.59 **	1.72 ± 1.96 **	0.14
4-month follow-up	3.24 ± 2.12	4.91 ± 8.28	0.45
Fibrinogen [g/L]			
At baseline	4.15 ± 0.9	3.49 ± 0.95	0.64
After rehabilitation	3.42 ± 0.64	2.77 ± 0.67 *	0.02
2-month follow-up	3.59 ± 0.72	2.94 ± 0.63	0.009
4-month follow-up	3.46 ± 0.68 ***	2.99 ± 0.45	0.39
Antithrombin III [%]			
At baseline	100.57 ± 12.19	94 ± 15.18	0.97
After rehabilitation	98.71 ± 9.59	90.14 ± 14.17	0.88
2-month follow-up	98.71 ± 8.68	92.14 ± 13.72	0.75
4-month follow-up	97.43 ± 7.19	92.86 ± 11.95	0.82
Vascular endothelial growth factor [pg/mL]			
At baseline	96.66 ± 28.47	79.79 ± 61.71	0.14
After rehabilitation	82.85 ± 24.87	57.62 ± 39.39	0.008
2-month follow-up	65.2 ± 31.65 **	63.67 ± 54.85	0.69
4-month follow-up	72.65 ± 41.13	52.98 ± 49.18 ***	0.067
Angiopoietin-2 [pg/mL]			
At baseline	1454.37 ± 467.35	2089.77 ± 922.54	0.78
After rehabilitation	954.4 ± 883.15	2128.25 ± 867.93	0.003
2-month follow-up	1517.2 ± 754.65	1865.6 ± 886.71	0.34
4-month follow-up	2131.93 ± 986.26 ***	5387.14 ± 805.20	0.23
NT-proBNP [pg/mL]			
At baseline	305.14 ± 197.96	214.29 ± 162.94	0.35
After rehabilitation	256.43 ± 236.33	81.71 ± 30.76 *	0.12
2-month follow-up	177 ± 197.47 **	81.86 ± 13.14 **	0.07
4-month follow-up	143.57 ± 127.46 ***	82.43 ± 32.97 ***	0.16

baPWV: ankle–shoulder pulse wave velocity; cfPWV_calc: carotid–femoral pulse wave velocity calculated from baPWV. *—when *p* < 0.05 for comparison between two indices: after rehabilitation and at baseline. **—when *p* < 0.05 for comparison between two indices: 1-month follow-up and at baseline. ***—when *p* < 0.05 for comparison between two indices: 2-month follow-up and at baseline.

**Table 5:** Parameters assessing quality of life.

Time Points	Control	Experimental Group	*p*-Value
Change in quality of life according to WHOQOL-BREF questionnaire (points)			
At baseline	3.29 ± 0.47	3.57 ± 0.51	0.06
After rehabilitation	3.57 ± 0.51	4.14 ± 0.36 *	0.022
2-month follow-up	3.57 ± 0.76	4.14 ± 0.66	0.06
4-month follow-up	3.71 ± 0.73 ***	4 ± 0.78	0.037
Satisfaction with the state of health according to WHOQOL-BREF questionnaire (points)			
At baseline	2.71 ± 0.47	3 ± 0.55	0.28
After rehabilitation	3 ± 0.55	3.57 ± 0.51 *	0.029
2-month follow-up	3 ± 0.96	3.57 ± 0.76 **	0.07
4-month follow-up	3.14 ± 0.66 ***	3.29 ± 0.73	0.59
Change in quality of life in terms of somatic domain according to WHOQOL-BREF questionnaire (points)			
At baseline	55.43 ± 10.07	53.57 ± 6.74	0.80
After rehabilitation	61.71 ± 11.15	54.57 ± 7.56	0.08
2-month follow-up	56.43 ± 8.61	58.14 ± 7.63	0.59
4-month follow-up	56.29 ± 6.09	55.71 ± 8.94	0.80
Change in quality of life in terms of psychological domain according to WHOQOL-BREF questionnaire (points)			
At baseline	61.71 ± 12.17	64.43 ± 11.28	0.53
After rehabilitation	63.43 ± 11.22	65.29 ± 8.39	0.66
2-month follow-up	63.43 ± 11.7	65.29 ± 8.39	0.87
4-month follow-up	63.43 ± 12.16	65.29 ± 8.5	0.73
Change in quality of life in terms of social domain according to WHOQOL-BREF questionnaire (points)			
At baseline	75 ± 12.97	74 ± 20.37	0.42
After rehabilitation	72.29 ± 8.35	67 ± 24.12 *	0.59
2-month follow-up	76 ± 10.57	66.86 ± 21.09 **	0.28
4-month follow-up	71.57 ± 15.75	65.29 ± 23.43 ***	0.47
Change in quality of life in terms of environmental domain according to WHOQOL-BREF questionnaire (points)			
At baseline	67.86 ± 13.35	70.71 ± 4.36	0.87
After rehabilitation	70.57 ± 16.46	72.43 ± 3.08	0.80
2-month follow-up	67 ± 14.7	72.57 ± 7.64	0.20
4-month follow-up	67.86 ± 15.35	70.71 ± 4.36	0.94

*—when *p* < 0.05 for comparison between two indices: after rehabilitation and at baseline. **—when *p* < 0.05 for comparison between two indices: 2-month follow-up and at baseline. ***—when *p* < 0.05 for comparison between two indices: 4-month follow-up and at baseline.

## Data Availability

Data are available on special and reasonable request.
